# Socio-Behavioural Barriers to Viral Suppression in the Older Adult Population in Rural South Africa

**DOI:** 10.1007/s10461-024-04328-9

**Published:** 2024-04-15

**Authors:** Chido Chinogurei, J. Manne-Goehler, K. Kahn, C. W. Kabudula, M. Cornell, J. K. Rohr

**Affiliations:** 1 Centre of Infectious Disease Epidemiology and Research, School of Public Health, University of Cape Town, Cape Town, South Africa; 2 Division of Infectious Diseases, Brigham and Women’s Hospital, Harvard Medical School, Boston, USA; 3 MRC/Wits Rural Public Health and Health Transitions Research Unit (Agincourt), School of Public Health, Faculty of Health Sciences, University of the Witwatersrand, Johannesburg, South Africa; 4 INDEPTH Network, Accra, Ghana; 5 Center for Population and Development Studies, Harvard University, Cambridge, MA USA

**Keywords:** Viral suppression, Older adults, Detectable viral load, Socio-behavioral, Internalized stigma

## Abstract

South Africa has the largest share of people living with HIV in the world and this population is ageing. The social context in which people seek HIV care is often ignored. Apart from clinical interventions, socio-behavioural factors impact successful HIV care outcomes for older adults living with HIV. We use cross-sectional data linked with demographic household surveillance data, consisting of HIV positive adults aged above 40, to identify socio-behavioural predictors of a detectable viral load. Older adults were more likely to have a detectable viral load if they did not disclose their HIV positive status to close family members (aOR 2.56, 95% CI 1.89-3.46), resided in the poorest households (aOR 1.98, 95% CI 1.23-3.18), or were not taking medications other than ART (aOR 1.83, 95% CI 1.02-1.99) likely to have a detectable. Clinical interventions in HIV care must be supported by understanding the socio-behavioural barriers that occur outside the health facility. The importance of community health care workers in bridging this gap may offer more optimum outcomes for older adults ageing with HIV.

## Introduction

The population of people living with HIV (PLHIV) in South Africa is aging. In 2012, HIV prevalence among individuals aged 50 years and older was 7.6%, which doubled in 2022 and is estimated to be > 22% by the end of 2030 [1–3]. This trend in aging with HIV is driven in part by widespread access to antiretroviral therapy (ART) that has transformed HIV into a chronic disease. There is robust evidence that early ART initiation has important impacts on health, especially as people age [4–7]. The third UNAIDS 95-95-95 target of viral suppression also hinges upon successful HIV management. Sustained viral suppression and continuous retention in care help to improve the quality of life amongst PLHIV [8, 9].

There are several studies that have looked at socio-behavioural barriers to viral suppression among vulnerable groups [10–13]. However, there are limited studies to understand how older adults in rural South Africa fare in terms of viral suppression and the socio-behavioural barriers which may impact the achievement of viral suppression. Although older adults have higher rates of viral suppression compared to the younger age-groups in South Africa, rates are still below the UNAIDS targets of 95%, with women being more virally suppressed than men [1, 2, 14].

HIV prevalence for adults aged between 15 and 49 is higher in urban areas than rural areas [15, 16]. As people age, they may migrate to the rural areas [17], where structural and patient-level barriers to optimal HIV care may be different compared to those in the urban areas [18, 19]. The rural setting in sub-Saharan Africa is often associated with limited economic opportunities and a lack of a retirement cushion in old age, which can have implications for retention in care [20]. Previous studies have reported barriers to viral suppression including low wealth, internal stigma, distance from the health facility, low educational attainment, and lack of geographic mobility [13, 21]. Socio-behavioural attributes for PLHIV may help us to understand why some individuals are virally suppressed or not. Retention in HIV care has been shown to be more difficult in rural compared with urban residences [22]; and also in households with lower socio-economic status [23]. Health behaviours have also been found in other studies to impact HIV care, including smoking and diet [24]. Social and behavioural factors may play an important role in the way older PLHIV manage their HIV care [25, 26].

In this study, we examined socio-behavioural factors associated with viral suppression amongst older PLHIV in a rural area of north-eastern South Africa, to identify barriers to successful long-term HIV management.

## Methods

### Study Population and Data

We used cross-sectional data from the Health and Aging in Africa: A Longitudinal Study of an INDEPTH community in South Africa (HAALSI). This study is a population-based survey focusing on men and women aged 40 years and older. The survey collects information on aging including health, physical and cognitive function, cardiometabolic health, economic well-being and HIV. The study population was nested within the Agincourt Health and socio-demographic surveillance system (HDSS) located in the rural north-eastern part of South Africa in Mpumalanga. The primary data used for this analysis came from two sources: data from HAALSI Wave 1 collected in 2015 and linked data from the Agincourt HDSS census on individual and household characteristics.

In total 5059 participants were included in the HAALSI baseline survey, 93% of whom consented to dry blood spot (DBS) biomarker testing in baseline survey. The survey and biomarker data were collected during in-person interviews by trained local fieldworkers using Computer Assisted Personal Interviews. Fieldworkers collected blood via finger prick and prepared DBS to assess participants’ HIV status, presence of ART in the bloodstream and viral load. HIV screening and confirmatory enzyme-linked immunosorbent assays (ELISA) were done using the Vironostika HIV 1/2 Ag/Ab MicroELISA System (BioMérieux, Marcy-l’Étoile, France) and the Roche CobasE411 Combi Ag, respectively [27]. The Viral Load Platform was Biomeriux NucliSens with a lower detection limit of < 100 copies by DBS. Our study was limited to participants who tested HIV positive and had a valid viral load measurement.

Ethical approvals for the study were granted from the University of the Witwatersrand Human Research Ethics Committee (ref. M141159), the Harvard T.H. Chan School of Public Health, Office of Human Research Administration (ref. C13–1608–02) and the Mpumalanga Provincial Research and Ethics Committee. Data for HAALSI is available upon request through the Harvard Dataverse repository.

### Study Variables

We defined viral suppression as a measure of ≤ 1000 copies/mL from DBS [28]. We obtained socio-demographic information including sex, marital status and age. From the HAALSI baseline survey, we also used self-reported disclosure of HIV status to close family members, self-reported use of herbal medication and self-reported medications usage. Self-reported disclosure of HIV status to family members was aggregated by combining questions on disclosure of status to siblings, spouse, parents or any other family members. The use of herbal medication was self-reported for several conditions including hypertension, diabetes, tuberculosis (TB), HIV, stroke and pain. We included self-reported current use of medications for TB, hypertension, diabetes, stroke, heart failure and chest pain to determine polypharmacy.

Household size, household access to government grants, socio-economic status and household road distance to the nearest health care facility were obtained from the linked Agincourt HDSS data. Socio-economic status (wealth index) was derived from a standard aggregation of construction materials of the main dwelling, type of toilet facilities, sources of water and energy, ownership of modern assets and livestock, employing the same methodology as implemented in the Demographic and Health Surveys [29, 30].

### Analysis

Viral suppression was the main outcome variable. We used the chi-2 test to assess if there was an association between viral suppression and participants’ demographic and socio-behavioural characteristics. We additionally employed logistic regression to examine the strength of the relationships between viral suppression and demographic as well as socio-behavioural attributes. The contextual variables and the adapted conceptual framework to guide this study are shown in Supplementary Information Figure I [31]. We used STATA version 14.2 for this analysis.

A sensitivity analysis was conducted to assess whether the results were influenced by viral load cut-off point and restricting to participants who were aware of their HIV positive status (Supplementary Information Table I and II). We used a cut-off of 200 copies/mL. Knowledge of HIV positive status was determined by either self-reporting of being HIV positive or ART being detected in the blood at the time of the study.

**Table 1:** Individual characteristics for demographic, household attributes and covariates of HAALSI participants living with HIV by viral suppression

	Undetectable viral load (*N* = 731)	Detectable viral load (*N* = 313)
*Sex*		
Male	321 (66.7%)	160 (33.3%)
Female	410 (72.8%)	153 (27.2%)
*Age group*		
40–49	197 (64.8%)	107 (35.2%)
50–59	263 (69.4%)	116 (30.6%)
60+	271 (75.1%)	90 (24.9%)
*Marital status*		
Married/Cohabiting	297 (71.4%)	119 (28.6%)
Separated/Divorced	142 (67.3%)	142 (32.7%)
Widowed	245 (72.5%)	93 (27.5%)
Never married	47 (59.5%)	32 (40.5%)
Household size (**)		
Single	55 (56.7%)	42 (43.3%)
2–4	195 (71.7%)	77 (28.3%)
5–7	249 (77.0%)	76 (23.1%)
8+	232 (66.3%)	118 (33.7%)
Socioeconomic status (*)		
Lowest	157 (62.1%)	96 (37.9%)
Lower	156 (72.6%)	59 (27.4%)
Middle	152 (70.7%)	63 (29.3%)
Rich	142 (71.7%)	56 (28.3%)
Upper	124 (76.1%)	39 (23.9%)
Distance to nearest health care facility (kms)	3.4 (2.4)	3.4 (2.6)
Household members awareness of HIV status (**)		
Yes	357 (81.1%)	83 (18.9%)
No	374 (61.9%)	230 (38.1%)
Households receiving any government grant (*)		
Yes	604 (72.0%)	235 (28.0%)
No	127 (62.0%)	78 (38.1%)
Polypharmacy (*)		
None	501 (67.0%)	247 (33.0%)
At least 1	230 (77.7%)	66 (22.3%)
*Herbal medication*		
Yes	72 (82.8%)	15 (17.2%)
No	659 (68.9%)	298 (31.1%)

Variable names with (*) and (**) implies significant chi square p-value at 5% and 1% respectively. Data are number (%) or mean (SD)

**Table 2:** Odd ratio coefficients from the logistic model of a detectable viral load among HAALSI participants on household attributes and other covariates by gender

	Crude OR (95% CI) *N* = 1044	Adjusted OR (95% CI) *N* = 1044
*Sex*		
Male	1.34 (1.02–1.74)*	1.38 (1.01–1.90)*
Female	1	1
*Age group*		
40–49	1.64 (1.17–2.29)**	1.61 (1.10–2.37)*
50–59	1.33 (0.96–1.84)	1.33 (0.93–1.90)
60+	1	1
*Marital status*		
Never married	1.79 (1.08–2.98)*	1.11 (0.63–1.96)
Married/Cohabiting	1.05 (0.77–1.45)	0.91 (0.63–1.31)
Separated/Divorced	1.28 (0.88–1.86)	1.09 (0.72–1.64)
Widowed	1	1
*Household size*		
Single	2.54 (1.58–4.11)**	1.52 (0.84–2.73)
2–4	1.32 (0.91–1.91)	1.22 (0.82–1.82)
5–7	1	1
8+	1.70 (1.21–2.39)**	2.02 (1.41–2.90)**
*Socio-economic status*		
Lowest	1.94 (1.25–3.02)**	1.98 (1.23–3.18)**
Lower	1.20 (0.75–1.92)	1.10 (0.67–1.81)
Middle	1.32 (0.83–2.10)	1.31 (0.80–2.13)
Rich	1.25 (0.78–2.02)	1.33 (0.81–2.20)
Upper	1	1
*Family members awareness of HIV status*		
Yes	1	1
No	2.65 (1.98–3.53)**	2.56 (1.89–3.46)**
*Household receiving any government grant*		
Yes	1	1
No	1.59 (1.15–2.17)**	1.26 (0.85–1.87)
*Household distance to nearest health facility*		
Less than 1 km	1	1
Between 1 km and 3kms	0.73 (0.49–1.10)	0.72 (0.47–1.11)
Between 3kms and 6kms	0.95 (0.63–1.42)	0.91 (0.59–1.40)
Over 6kms	0.85 (0.51–1.40)	0.88 (0.52–1.50)
*Polypharmacy*		
No	1.72 (1.25–2.35)**	1.43 (1.02–1.99)*
At least one	1	1
*Herbal medication*		
Yes	1	1
No	2.17 (1.22–3.85)**	1.83 (1.00-3.45)

Data with * and ** implies significant p-value for regression coefficient at 5% and 1% respectively

## Results

The prevalence of HIV was 23% (*n* = 1048). We obtained 1044 valid viral load values from the DBS results (Supplementary Information Figure II). Overall, 70% of the PLHIV had an undetectable viral load. The descriptive and bivariate analyses showing differences in viral suppression results and socio-behavioural characteristics are shown in Table 1. We found a significant association between viral suppression and various factors, including household size, socio-economic status, households receiving any state grant, household members’ awareness of an individual’s HIV status and polypharmacy. The odds of viral suppression were lowest in single-person households. Those with the lowest socio-economic status were less likely to achieve viral suppression than individuals in higher socio-economic groups. Failure to disclose HIV status to close family members was associated with a lower likelihood of viral suppression compared to those who had disclosed their status. Households not receiving any state grant were less likely to achieve viral suppression than those receiving one. Individuals solely on ART without additional medication were less likely to achieve viral suppression compared to those taking medication for at least one other condition besides HIV.

In the adjusted model for logistic regression, individuals in households with more than eight members had twice the odds of having a detectable viral load compared to those with a household size of between five and seven members (Table 2). Individuals living in households with the lowest socio-economic status had twice the odds of having a detectable viral load compared to those residing in households with higher socio-economic status. Respondents who reported that their close family members were unaware of their status had 2.6 times the odds of having a detectable viral load compared to those whose family members were aware of their HIV positive status. Disclosure to close family members had the strongest association in the adjusted model. Individuals solely taking ART without any additional medication had 43% higher odds of having a detectable viral load compared to those taking medication for at least one other condition besides HIV.

In the sensitivity analysis, when a lower viral suppression threshold was applied and when restricted to individuals who were aware of their HIV positive status, we found that PLHIV who were not using any herbal medication at the time of the study were significantly more likely to have a detectable viral load compared to those using herbal medication (Supplementary information Table I and II).

## Discussion

The study helps us understand why the setting underlying HIV clinical care is important in improving health outcomes and attaining better quality of life among older PLHIV. We were interested in understanding how socio-behavioural characteristics were associated with lower viral suppression outcomes for older PLHIV within the rural context of South Africa. We found that being male, aged between 40 and 49 years, and failing to disclose HIV status to close family members, were associated with detectable viral load. Additionally, larger and poor households and those not taking medication for other comorbid conditions apart from HIV were more vulnerable to lower suppression rates.

Our study provides additional evidence of lower suppression rates for males than females [1, 14, 32, 33]. Women often interact with the health care system more than men. This may be partly because women have unique health needs, such as reproductive health, and are more likely to seek preventive care [34]. In the context of HIV, prevailing notions of masculinity may act as obstacles to men accessing health care facilities [35]. Our youngest age group of older adults in this study, those aged between 40 and 49 years, were at the highest risk of not being virally suppressed. It is likely they may have been newly infected and may have taken longer to transition into HIV care, especially for the men [2, 36, 37]. Conversely, it may be due to selection effects of mortality in the higher age groups, those that are older and virally unsuppressed may have died [7].

The most significant factor contributing to an unsuppressed viral load was the failure to disclose one’s HIV status to close family members. Similar to a study in rural Uganda, non-disclosure of HIV status to other household members may hinder viral suppression amongst those older PLHIV [18]. This is more likely linked to fear that close family members will reject them once they reveal their HIV positive status. Similar findings also highlight the association between HIV care and internalised stigma [38–41]. Internalised stigma has been found in other studies to be associated with depression and other mental health issues [42–44]. Disclosure to close family members may help in strengthening HIV care interventions through the provision of emotional and in some cases financial support within the household [45–47]. The HIV care and treatment programs should also consider approaches to HIV care that may help PLHIV towards addressing some of the fears that inhibit them from sharing their status to close family members.

The study found that poverty was a barrier to attaining viral suppression. Other studies have found this to be related to costs incurred by patients in accessing the health care facility [48]. However, in this study, they are likely due to other factors since health care services are mostly in proximity and there is free access to community clinics. The effect of poverty has been linked to food security and inadequate nutrition in other studies [49–51]. This effect may be exaggerated in larger, inter-generational households due to the difficulty in prioritizing of scarce resources within the household. Inter-generational households have existed since apartheid mainly due to labour migration from tribal homelands. Parents would leave their children with grandparents in their care while they worked in urban areas [52]. The impact of HIV morbidity and mortality has also increased the number of inter-generational households [53, 54], as well increased unemployment in South Africa [55, 56]. The increase in unemployment may particularly force an increase in inter-generational households due to greater dependency on older members of the households. This may also aggravate challenges to health care utilization among older adults due to the prioritisation of resources elsewhere within the household.

Polypharmacy shows extended benefits for viral suppression which is likely a reflection of HIV care utilization, particularly for comorbidities which are monitored within the standard health care protocol [57, 58]. In the rural South African context, where herbal medicines are commonly used, it was intriguing to discover that individuals who supplemented ART with herbal medication experienced better outcomes than those who did not use herbal medications. These findings are unique in the context of rural care-seeking behaviour among older PLHIV. In other contexts, herbal medications are often taken for enhancing immunity and managing opportunistic infections among PLHIV [59, 60]. On the other hand, some studies found no clinical improvement in some herbal interventions and clinicians discourage concomitant use of traditional medicines with ART due to unfavourable effects of the medication interactions [61, 62]. The difference observed could also be a result of health seeking behaviour amongst those that use herbal medication. It may need further study to identify whether this is an effect resulting from better health awareness or the clinical impact of herbal medication.

In rural areas of South Africa, strong family and community bonds exist, and reducing internalised stigma will not only encourage individuals to seek family support for HIV-related health issues but will also extend support to those around the community. The findings underscore the necessity for integrated strategies to address internalised stigma among older individuals living with HIV. Such approaches not only enhance family support but also mitigate the mental health burden associated with internalized stigma. This will provide a holistic approach to both clinical and socio-behavioral well-being of older PLHIV. Expanding HIV programs with social workers and community health care workers within the rural communities may assist in this effort.

## Conclusion

While acknowledging its limitations, including the inability to establish causality from associations, this study sheds light on socio-behavioral barriers to HIV care frequently faced by older PLHIV. The research contributes to our comprehension of these socio-behavioral obstacles, which are crucial for achieving the UNAIDS 95-95-95 targets for viral suppression. These barriers are often overlooked in the clinical realm of HIV care. It is important to note that the study’s findings may not be broadly applicable to PLHIV in South Africa but may be a representation of rural South Africa. Collaborative efforts between community health care workers and practitioners are essential for strengthening initiatives aimed at enhancing HIV care outcomes and ensuring inclusivity. Integrating social and behavioral considerations into HIV care and treatment programs is imperative for reinforcing health policies. As the HIV population in South Africa continues to age due to improved access to ART, these findings provide valuable insights for guiding updates in future HIV care policies.
